# SlyA Transcriptional Regulator Is Not Directly Affected by ppGpp Levels

**DOI:** 10.3389/fmicb.2020.01856

**Published:** 2020-08-04

**Authors:** Julia Bartoli, Julie Pamela Viala, Emmanuelle Bouveret

**Affiliations:** ^1^LISM, Institut de Microbiologie de la Méditerranée, CNRS, Aix-Marseille University, Marseille, France; ^2^SAMe Unit, Microbiology Department, Pasteur Institute, Paris, France

**Keywords:** SlyA, ppGpp, *Escherichia coli*, stress response, *hlyE*

## Abstract

The SlyA transcriptional regulator controls the expression of genes involved in virulence and production of surface components in *S. Typhimurium* and *E. coli*. Its mode of action is mainly explained by its antagonism with the H-NS repressor for the same DNA binding regions. Interestingly, it has been reported that the alarmone ppGpp promotes SlyA dimerization and DNA binding at the promoter of *pagC*, enhancing the expression of this gene in *Salmonella*. A recurring problem in the field of stringent response has been to find a way of following ppGpp levels *in vivo* in real time. We thought that SlyA, as a ppGpp responsive ligand, was a perfect candidate for the development of a specific ppGpp biosensor. Therefore, we decided to characterize in depth this SlyA control by ppGpp. However, using various genes whose expression is activated by SlyA, as reporters, we showed that ppGpp does not affect SlyA regulation *in vivo*. In addition, modulating ppGpp levels did not affect SlyA dimerization *in vivo*, and did not impact its binding to DNA *in vitro*. We finally showed that ppGpp is required for the expression of *hlyE* in *E. coli*, a gene also activated by SlyA, and propose that both regulators are independently required for *hlyE* expression. The initial report of ppGpp action on SlyA might be explained by a similar action of SlyA and ppGpp on *pagC* expression, and the complexity of promoters controlled by several global regulators, such as the promoters of *pagC* in *Salmonella* or *hlyE* in *E. coli*.

## Introduction

SlyA is a transcriptional regulator that belongs to the MarR superfamily of regulators (Will and Fang, 2020). Since its discovery as an inducer of hemolytic activity (Libby et al., 1994), several genes have been shown to be regulated by SlyA in *Salmonella enterica* and in *Escherichia coli*, however their regulons are different in these two bacteria. In *Salmonella*, SlyA controls the expression of genes required for virulence (Navarre et al., 2005; Ellison and Miller, 2006). A *slyA* mutant is impaired for growth within macrophages and is hyper susceptible to oxidative stress (Ellison and Miller, 2006). In *E. coli*, SlyA activates the expression of the cryptic hemolysin *hlyE* (*clyA*) (Wyborn et al., 2004; Lithgow et al., 2007), of Type1 fimbriae (McVicker et al., 2011), of *pagP* involved in lipid A palmitoylation in biofilm (Chalabaev et al., 2014), and of K5 capsule gene cluster (Corbett et al., 2007). In addition to these reports on specific genes, a global study has recently expanded the proposed repertoire of the SlyA regulon in *E. coli*, with cryptic genes coding for potential fimbrial-like adhesins that contribute to biofilm formation (Curran et al., 2017). Furthermore, this latter study permitted the refinement of a SlyA binding motif in *E. coli*.

SlyA binds DNA as a dimer. It functions mainly as counter-silencer by antagonizing and displacing the H-NS repressor (Stoebel et al., 2008; Will et al., 2015). Interestingly, in *E. coli*, *slyA* expression is positively autoregulated, independently of H-NS (Corbett et al., 2007). However, the majority of SlyA targets reported so far are known or predicted to be repressed by H-NS (Curran et al., 2017). The condition of induction of *slyA* itself or the potential ligand molecule of SlyA have not been elucidated. SlyA has been crystalized with a bound salicylate molecule and it was shown *in vitro* that this binding inhibited SlyA binding to DNA (Dolan et al., 2011; Will et al., 2019).

It has been reported that ppGpp nucleotide promotes SlyA dimerization and binding to its target promoters in *Salmonella*, and that ppGpp is required for SlyA activity *in vivo* (Zhao et al., 2008). ppGpp is an important nucleotide acting as a secondary messenger of the stringent response (Potrykus and Cashel, 2008). This global stress response plays a central role in the physiology of bacteria, and its main role is to slow down ribosome biosynthesis and activity while promoting survival programs. This response has been the subject of a strong and renewed interest in the last years when its importance in pathogenicity and resistance to antibiotics has been (re)discovered (Dalebroux et al., 2010; Hobbs and Boraston, 2019). There are two main modes of action of ppGpp, whose relative importances depend on bacteria:in *E. coli* and closely related bacteria, ppGpp binds RNAP in conjunction with DksA, influencing globally the transcriptome landscape of the cell (Gourse et al., 2018). In addition, ppGpp inhibits enzymes of the guanosine synthesis pathway and ribosomal GTPases (Bennison et al., 2019). The possible allosteric regulation of SlyA by ppGpp triggered high interest at the time, as shown by its highlight in an important review discussing the role of ppGpp in virulence (Dalebroux et al., 2010). If validated, this behavior might have provided a good base for the design of direct ppGpp biosensors that are still missing in the field for live detection and/or imaging of ppGpp in bacteria. However, there has been no further mention of this result or follow-up in the literature. It was only mentioned in a discussion that ppGpp was not required for *fimB* activation by SlyA in *E. coli* (McVicker et al., 2011).

Therefore, we decided to study and characterize clearly this proposed role of ppGpp in controlling SlyA mechanism. The results presented here, based on a combination of genetics, molecular, and biochemical approaches, show that ppGpp is not directly involved in the molecular mechanism of SlyA dimerization and DNA binding. However, for some SlyA regulated genes (like *hlyE* in *E. coli* or *pagCD* in *Salmonella*), complex regulation network involving H-NS and other global regulators might explain indirect ppGpp effects.

## Materials and Methods

### Plasmid Constructions

Plasmid constructions are described succinctly in Table 1. The *slyA* ORFs from *E. coli* or *Salmonella enterica* s. Typhimurium 12023 were amplified by PCR on genomic DNA template using the indicated oligonucleotides and cloned in the pBAD24 (pEB227) and pET-6His-Tev vectors (pEB1188).

**TABLE 1:** Plasmids.

**Lab code**	**Name**	**Description**	**References**
pEB227	pBAD24	amp^R^, colE1 ori, PBAD promoter	Guzman et al., 1995
pEB1610	pBAD-SlyA_stm	PCR with primers ebm1026/1027 (EcoRI/XhoI) cloned in pBAD24 (EcoRI/SalI)	This work
pEB1609	pBAD-SlyA_eco	PCR with primers ebm1026/1189 (EcoRI/XhoI) cloned in pBAD24 (EcoRI/SalI)	This work
pEB0898	pUA66	kana^R^, pSC101 ori, GFPmut2	Zaslaver et al., 2006
pEB0987	pUA139	kana^R^, pSC101 ori, GFPmut2	Zaslaver et al., 2006
pEB1994	pUA-*paaA*	PCR with primers ebm1830/1831 cloned in pEB898 (XhoI/BamHI)	This work
pEB2005	pUA-*pagC*_Stm	PCR with primers ebm1847/1848 cloned in pEB898 (XhoI/BamHI)	This work
pEB2006	pUA-*pagD*_Stm	PCR with primers ebm1847/1848 cloned in pEB987 (BamHI/XhoI)	This work
pEB1937	pUA-*fimB*	PCR with primers ebm1755/1756 cloned in pEB898 (XhoI/BamHI)	This work
pEB1993	pUA-*elfA*	PCR with primers ebm1832/1833 cloned in pEB898 (XhoI/BamHI)	This work
	pUA-*slyA*	Transcriptional fusions available in the plasmid library described in the indicated reference.	Zaslaver et al., 2006
	pUA-*hlyE*		
	pUA-*pagP*		
	pUA-*agaS*		
	pUA-*ybeT*		
	pUA-*ssuE*		
	pUA-*yehD*		
	pUA-*ybeU*		
	pUA-*ygeG*		
	pUA-*agaS*		
	pUA-*ycjM*		
	pUA-*yadN*		
pEB1188	pET-6His-Tev		Wahl et al., 2011
pEB1885	pET-6His SlyA_stm	PCR with primers ebm1026/1027 cloned in pEB1188 (EcoRI/XhoI)	This work
pEB2004	pET-6His SlyA_eco	PCR with primers ebm1026/1189 cloned in pEB1188 (EcoRI/XhoI)	This work
pEB0267	pKD46	repA101(ts) Pbad-gam-bet-exo Ampi^R^	Datsenko and Wanner, 2000
pEB0794	pJL148	-SPA-FRT-kana^R^-FRT Ampi^R^	Zeghouf et al., 2004
pEB0266	pCP20	pSC101(ts), encoding FLP gene, Ampi^R^, Cam^R^	Cherepanov and Wackernagel, 1995
pEB0697	pALS10	P*tac-relA*, Ampi^R^	Svitil et al., 1993
pEB0698	pALS13	P*tac*-*relA*(1–455), Ampi^R^	Svitil et al., 1993
pEB0699	pALS14	P*tac*-*relA*(1–331), Ampi^R^	Svitil et al., 1993

Transcriptional fusions with GFP were constructed in the pUA66 (pEB898) or pUA139 (pEB987) vector backbone (Zaslaver et al., 2006). When available, transcriptional fusions were retrieved from the Zaslaver collection (Zaslaver et al., 2006), or else the promoter regions were PCR amplified using the oligonucleotides listed in Table 2 and cloned between XhoI and BamHI restriction sites. The Ecocyc website (Karp et al., 2018) was used for sequence retrieval.

**TABLE 2:** Oligonucleotides.

**Lab Code**	**5′ –>3′ sequence**	**Gene**
Ebm623	GCCCTTTCGTCTTCACCTCG	FW promoters
Ebm629	ATCTCCTTCTTAAATCTAGAGGATC	RV promoters
Ebm1830	CCGCTCGAGTCGCTACTCTCCAGATGTTTCAC	PpaaA FW
Ebm1831	ACGGGATCCTCAAAGCGTTCTTCTTGGGTCAC	PpaaA RV
Ebm1847	CATCTCGAGATGATGTTTCATAGCACCTCCTG	PpagC FW
Ebm1848	ACGGGATCCTAGCACGCTTTATTCCCGCTCC	PpagC RV
Ebm1755	CCGCTCGAGTGCGTTCCCCCATATCTCTAGG	PfimB FW
Ebm1756	ACGGGATCCCCATGCTCTTGCATGCTATGTACC	PfimB RV
Ebm1832	CCGCTCGAGATTCCAGCAAGGAGCTGGAGC	PelfA FW
Ebm1833	ACGGGATCCACGCTGGACGTTGCACATACC	PelfA RV
Ebm1026	ACCGAATTCTTGGAATCGCCACTAGGTTCTG	slyA FW
Ebm1189	ACGCTCGAGTCACCCTTTGGCCTGTAACTC	slyA_coli RV
Ebm1027	TTGCTCGAGTCAATCGTGAGAGTGCAATTCC	slyA_salmo RV
Ebm1855	ATCGCAAAACTTGAGCATAATATCATTGAGTTA CAGGCCAAAGGGATTCCAACTACTGCTAGC	slyA-3Flag FW
Ebm1856	TAAGTTTGCGTGTGGTCAGGTTACTGACCACA CGCCCCCTTCATTCATATGAATATCCTCCTTAG	slyA-3Flag RV

### Strain Constructions

The construction of the various strains is described succinctly in Table 3. Insertion of the 3Flag sequence in fusion with the *slyA* ORF on the chromosome was done by direct recombination of a PCR fragment amplified with oligonucleotides ebm1855/1856 and pJL148 plasmid (Zeghouf et al., 2004) as template, following the Datsenko and Wanner procedure (Datsenko and Wanner, 2000). Deletion mutant alleles obtained from the Keio collection (Baba et al., 2006) or tagged alleles obtained by recombination were transduced from one genetic background to another by generalized transduction with phage P1. The kanamycin resistance cassette was removed by transformation with the pCP20 plasmid (Cherepanov and Wackernagel, 1995).

**TABLE 3:** Strains.

**Lab code**	**Name**	**Description**	**References**
EB072	BL21(DE3)pLys	Coli B λ(DE3) pLysS(cmR)	Studier and Moffatt, 1986
EB240	BW25113Δ*slyA*	Δ*slyA*::kana^R^	Baba et al., 2006
EB126	BW25113Δ*relA*	Δ*relA*::kana^R^	Baba et al., 2006
EB559	MG1655Δ*dksA*		Wahl et al., 2011
EB761	BW25113Δ*cyaA*	Δ*cyaA*::kana^R^	Baba et al., 2006
EB128	BW25113Δ*fis*	Δ*fis*::kana^R^	Baba et al., 2006
EB047	BW25113Δ*hns*	Δ*hns*::kana^R^	Baba et al., 2006
EB944	MG1655	Wild type reference. F- λ- rph-1	Lab stock
EB425	MG1655 ppGpp°	Δ*relA*Δ*spoT*::cat	Wahl et al., 2011
EB1073	MGΔ*slyA*	P1 transduction Δ*slyA*::kana^R^ from	This work
		EB240 to EB944. Kanamycin resistance removed with pCP20	
EB1076	MGΔ*slyA*Δ*relA*	P1 transduction Δ*relA*::kana^R^ from EB126 to EB1073. Kanamycin resistance removed with pCP20	This work
EB1077	ppGpp°_Δ*slyA*	P1 transduction Δ*spoT*::*cat* from EB425 to EB1076	This work
EB1100	Δ*dksA* Δ*slyA*	P1 transduction Δ*slyA*::kana^R^ from EB240 to EB559. Kanamycin resistance removed with pCP20	This work
EB781	MGΔ*cyaA*	P1 transduction Δ*cyaA*::kana^R^ from EB761 to EB944. Kanamycin resistance removed with pCP20	This work
EB743	MGΔ*fis*	P1 transduction Δ*fis*::kana^R^ from EB128 to EB944. Kanamycin resistance removed with pCP20	This work
EB951	MGΔ*hns*	P1 transduction Δ*hns*::kana^R^ from EB047 to EB944. Kanamycin resistance removed with pCP20	This work
EB1106	*MG_slyA*-3Flag	PCR ebm1855/1856 on pJL148, λRed recombination in EB944 followed by P1 transduction in EB944	This work
EB468	MG_ppGpp°	P1 transduction slyA-3Flag-kana^R^ from EB1106 to EB425	This work
	SlyA-3Flag		

### Measure of Expression Using Transcriptional Fusions With GFP

*Escherichia coli* strains were transformed by the plasmids carrying the GFP transcriptional fusions, with or without pBAD plasmids producing SlyA proteins, and the selection plates were incubated at 37°C for 16 h. 600 μl of LB medium supplemented with the required antibiotics, and 0.05% arabinose for pBAD induction, were inoculated (three to six replicates for each assay) and grown for 16 h at 30°C in 96-well polypropylene plates of 2.2-ml wells under aeration and agitation. Fluorescent intensity measurement was performed in a Tecan infinite M200 plate reader. 150 μl of each well was transferred into a black Greiner 96-well plate for reading optical density at 600 nm (OD600) and fluorescence (excitation, 485 nm; emission, 530 nm). The expression levels were calculated by dividing the intensity of fluorescence by the OD600. After mean values were calculated, values from the control vector were subtracted. The results are given in arbitrary units, because the intensity of fluorescence is acquired with an optimal and variable gain; hence, the absolute values cannot be compared between different panels. The error bars on the figures show the standard error of the mean (SEM).

### Purification of SlyA Proteins

BL21(DE3)pLysS strain was transformed with plasmids pET6HisTev-*slyA_stm* (pEB1885) or pET6HisTev-*slyA_ecoli* (pEB2004). The strains were grown in 500 ml LB at 30°C. At OD_600nm_ = 0.9, 1 mM IPTG was added and the cultures incubated during 6 h at 23°C. The proteins were then purified following the procedure described previously (Wahl et al., 2011).

### Electrophoretic Mobility Shift Assay

DNA fragments containing the *pagC_stm*, *hlyE*, or *slyA* promoters were obtained by PCR using the corresponding transcriptional fusion plasmids as matrices, and the ebm623 and ebm629 primers that hybridize at the limit of the cloning sites. The PCR fragments were then purified using Macherey Nagel PCR purification kit. 20 nM PCR fragments were incubated with purified SlyA and ppGpp (TriLink Biotechnologies) (see legends of Figure 4 and Supplementary Figure S3 for the concentrations), in a 20 μl final reaction buffer containing 25 mM Tris–HCl (pH 7.2), 10 mM MgCl_2_, 1 mM CaCl_2_, 0.5 mM EDTA, 50 mM KCl, and 5% glycerol. The mix was incubated for 30 min at 20°C. The reactions were then analyzed by native PAGE (Acrylamide 10% 29:1). DNA was stained with GelRed (Fluo-Probes).

### *In vivo* Crosslinking With Formaldehyde

Cells were pelleted and washed once with 10 mM potassium phosphate buffer, pH 6.8, and resuspended in the same volume, with (+F) or without (-F) formaldehyde 1%. Samples were incubated for 15 min at room temperature. The cells were then pelleted and washed again before solubilization in Laemmli loading buffer (volume normalized according to the OD_600_ of the initial cultures). Before loading on SDS-PAGE, the samples were either heated 10 min at 37°C to maintain the cross-links, or heated 20 min at 96°C to destroy them. SDS-PAGE, electrotransfer onto nitrocellulose membranes, and Western blot analyses were performed as previously described (Bouveret et al., 1995). The monoclonal anti-M2 Flag antibody used for 3Flag tag detection was purchased from Sigma.

## Results

**FIGURE 1:**
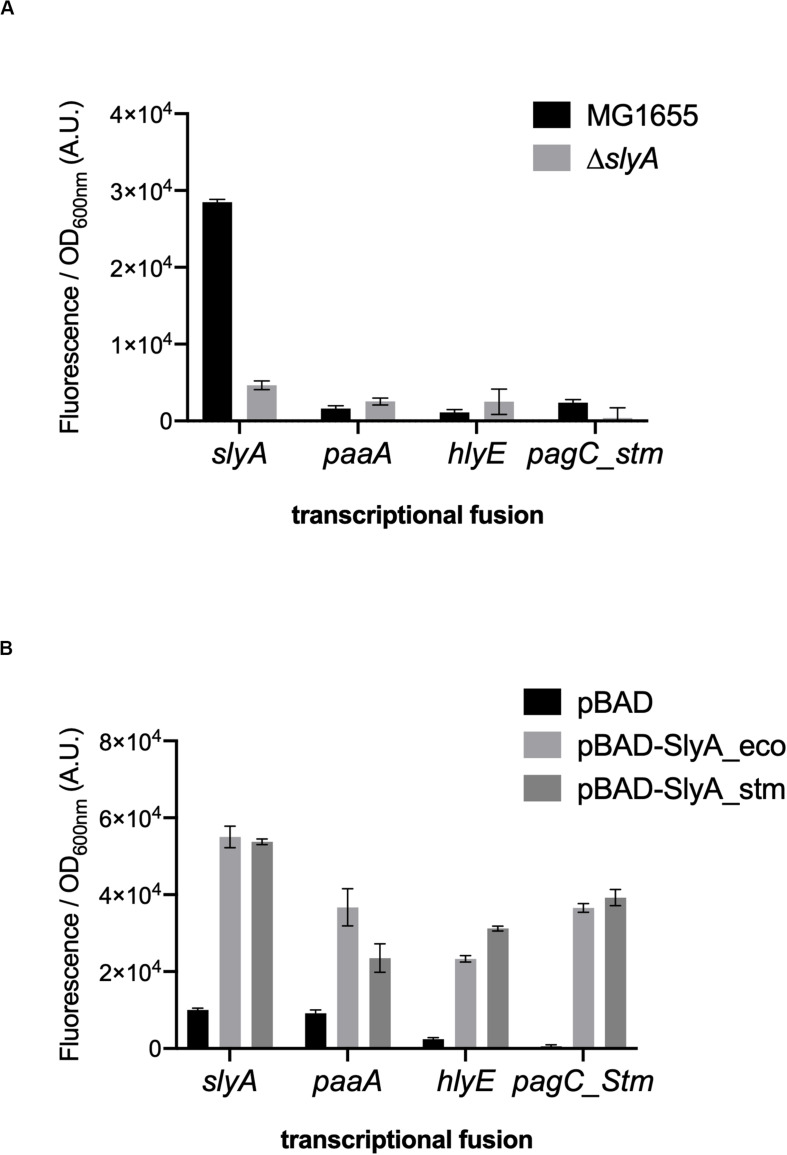
*slyA*, *paaA*, *hlyE*, and *pagC*_stm promoters are induced by SlyA. **(A)** Comparison of transcriptional fusion activity in wild type MG1655 and in the *slyA* mutant EB1073 strains grown overnight at 30°C in LB. **(B)** Transcriptional fusion activity when SlyA protein is overproduced. MG1655 strains transformed by the indicated transcriptional fusions and the pBAD24 (pEB227), pBAD-slyA_ecoli (pEB1609), or pBAD_slyA_stm (pEB1610) plasmids were incubated overnight at 30°C in LB supplemented with 0.05% arabinose. The activities correspond to the ratio between GFP fluorescence and OD_600nm_ of 6 replicates, given in arbitrary units (A.U.). The error bars show the SEM.

We wanted to study the effect of ppGpp in the activation of gene expression by SlyA in *E. coli*. In addition to the known SlyA targets *hlyE*, *fimB*, and *slyA* itself, it was reported that SlyA might influence the expression of many genes when overexpressed (Curran et al., 2017). Based on this study, we tested a set of transcriptional fusions to select the ones that will allow us to follow the activity of SlyA. We used transcriptional fusions with GFP already available in a published *E. coli* promoter library (Zaslaver et al., 2006):*pagP*, *slyA*, *hlyE*, *agaS*, *ybeT*, *ssuE*, *yehD*, *ybeU*, *ygeG*, *agaS*, *ycjM*, and *yadN*. We completed this set by constructing transcriptional fusions missing in the library with the promoters of *elfA*, *fimB*, and *paaA* of *E. coli*, and also with promoters of *Salmonella pagC* and *pagD*, which are regulated by the SlyA/H-NS antagonism and reported to be affected by ppGpp (Zhao et al., 2008). We then measured the expression of all these transcriptional fusions in wild type and Δ*slyA* strains (Figure 1A and Supplementary Figure S1A), as well as in the Δ*slyA* strain overproducing or not SlyA from a pBAD inducible plasmid (Figure 1B and Supplementary Figure S1B). From this, we selected 4 reporters that responded robustly to SlyA: *slyA* itself, *paaA*, *hlyE*, and *pagC*_Stm (Figure 1 and Supplementary Figure S1).

**FIGURE 2:**
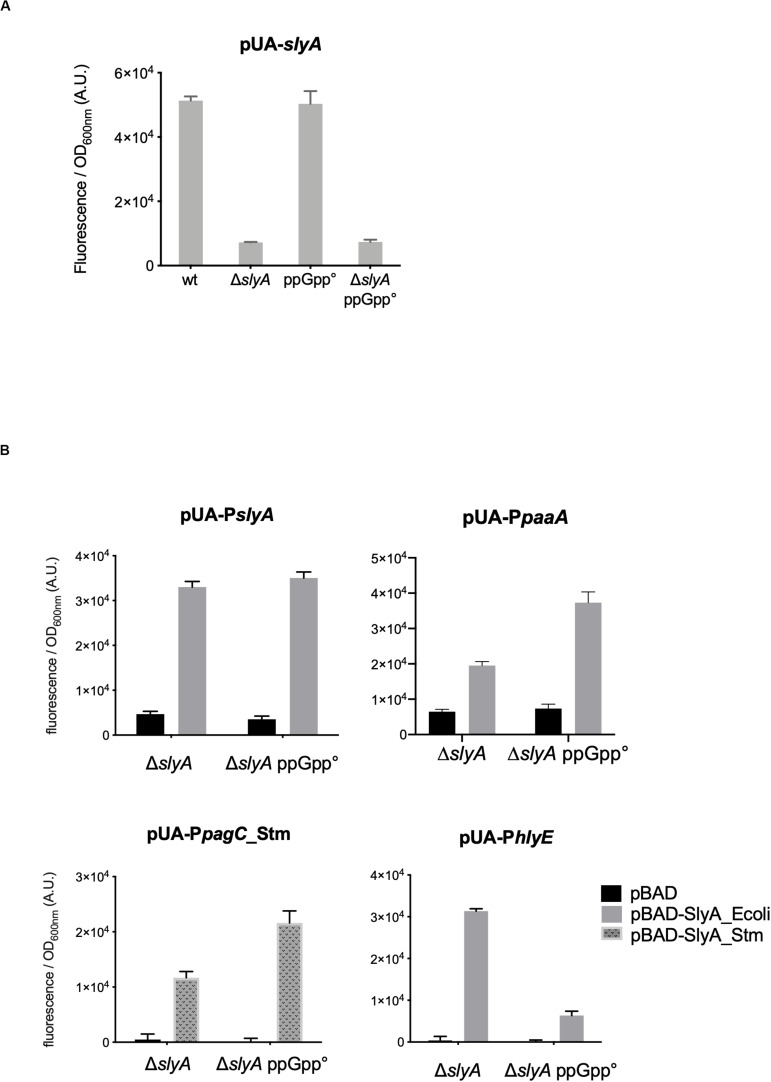
*In vivo* induction by SlyA does not require ppGpp. **(A)** Comparison of *slyA* transcriptional fusion activity in wild type MG1655, Δ*slyA* (EB1073), ppGpp° (EB425), and Δ*slyA_*ppGpp° (EB1077) strains grown overnight at 30°C in LB. **(B)** Effect of pBAD-SlyA overproduction in Δ*slyA* or Δ*slyA_*ppGpp° strains on the expression of *slyA*, *paaA*, *pagC*_Stm, and *hlyE* transcriptional fusions, in the same conditions as in Figure 1B. The activities correspond to the ratio between GFP fluorescence and OD_600nm_ of six **(A)** or four **(B)** replicates, given in arbitrary units (A.U.). The error bars show the SEM.

The *slyA* transcriptional fusion was the only one to show a strong expression level in the wild type strain (Figure 1A). We therefore compared its expression in strains devoid of ppGpp (strains deleted of the *relA* and *spoT* genes). The absence of ppGpp did not modify the expression of *slyA* (Figure 2A). We then compared the expression of the four transcriptional fusions selected above in Δ*slyA* strains overproducing SlyA, in the presence or in the absence of ppGpp (Figure 2B). For the *slyA*, *paaA*, and *pagC*_Stm fusions, the absence of ppGpp did not prevent the induction by SlyA. These results indicate that *in vivo*, ppGpp is not required for the mechanism of transcription activation by SlyA.

**FIGURE 3:**
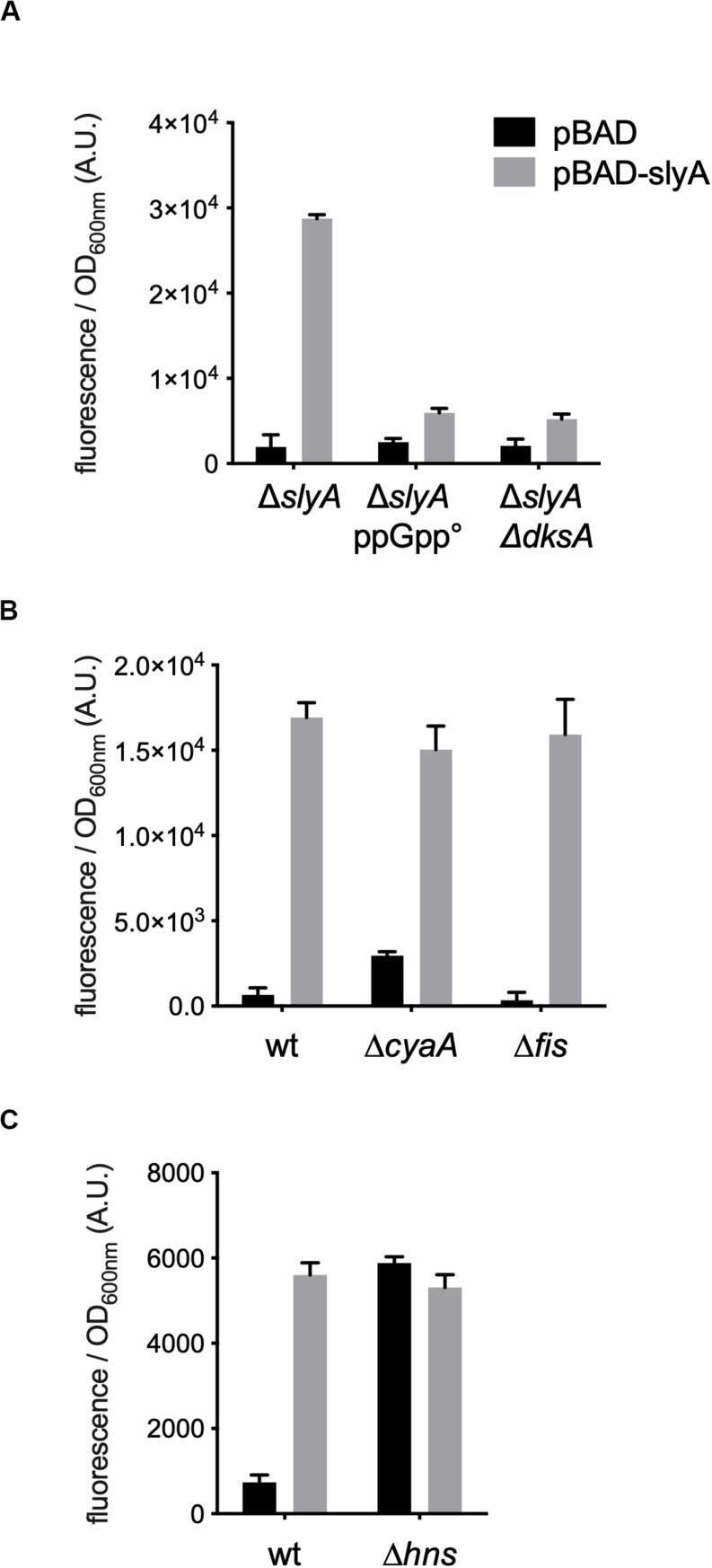
*hlyE* activation by SlyA in global regulatory mutants. Induction of the *hlyE* transcriptional fusion by pBAD-slyA_ecoli was tested in the same conditions as in Figure 1B, in the indicated mutant strains: wt (MG1655), Δ*slyA* (EB1073), Δ*slyA_*ppGpp (EB1077), Δ*slyA*Δ*dksA* (EB1100), Δ*cyaA* (EB781), Δ*fis* (EB743), and Δ*hns* (EB951). The activities correspond to the ratio between GFP fluorescence and OD_600nm_ of 4 **(A,C)** or 3 **(B)** replicates, given in arbitrary units (A.U.). The error bars show the SEM.

However, the induction of the *hlyE* fusion by SlyA was strongly decreased in the absence of ppGpp (Figure 2B). To characterize better the specific effect of ppGpp on *hlyE*, we tested its induction by SlyA in different mutants for global regulatory factors. First, we tested the action of SlyA in the Δ*dksA* mutant. DksA is a cofactor of the RNA polymerase, required for the regulation of RNAP by ppGpp (Gourse et al., 2018). While ppGpp is still present in this mutant, *dksA* deletion mimics the global effects of a ppGpp° mutant on gene transcription due to the action of ppGpp on RNA polymerase. While SlyA still activated the expression of the *slyA* transcriptional fusion in the Δ*dksA* mutant, as in the ppGpp° mutant (Supplementary Figure S2), SlyA induction of *hlyE* was strongly decreased in the Δ*dksA* mutant, similarly to what was observed in the ppGpp° mutant (Figure 3A). This result suggests that the effect of ppGpp on *hlyE* expression is due to its role in controlling expression through RNAP regulation (at *hlyE* promoter or others), and not to a direct control of SlyA activity. For full activation of its expression, *hlyE* would therefore need both SlyA overproduction (or activation by unknown conditions), and the presence of ppGpp.

In addition to SlyA, *hlyE* expression is controlled by a network of global regulators, such as H-NS (Wyborn et al., 2004; Lithgow et al., 2007), CRP-cAMP and FNR (Westermark et al., 2000), and it was also reported that it is negatively regulated by Fis (Bradley et al., 2007). ppGpp is also a member of this complex network controlling bacterial physiology (Travers and Muskhelishvili, 2005). Therefore the ppGpp/DksA effect observed on the expression of *hlyE* might be indirect through one or several of these global regulators. We tested *hlyE* induction by SlyA in *hns*, *fis*, and *cyaA* mutants. SlyA was still able to induce *hlyE* expression in *fis* and *cyaA* mutants (Figure 3B). As expected, the expression of hlyE was de-repressed in the Δ*hns* mutant, and not further induced by the presence of SlyA (Figure 3C). This confirmed that SlyA activation of *hlyE* expression is due to the displacement of H-NS. This set of experiments suggests that ppGpp role in *hlyE* expression is not due to an indirect effect through CRP-cAMP or Fis regulators, but probably through the regulation of RNAP at the *hlyE* promoter in synergy with DksA.

**FIGURE 4:**
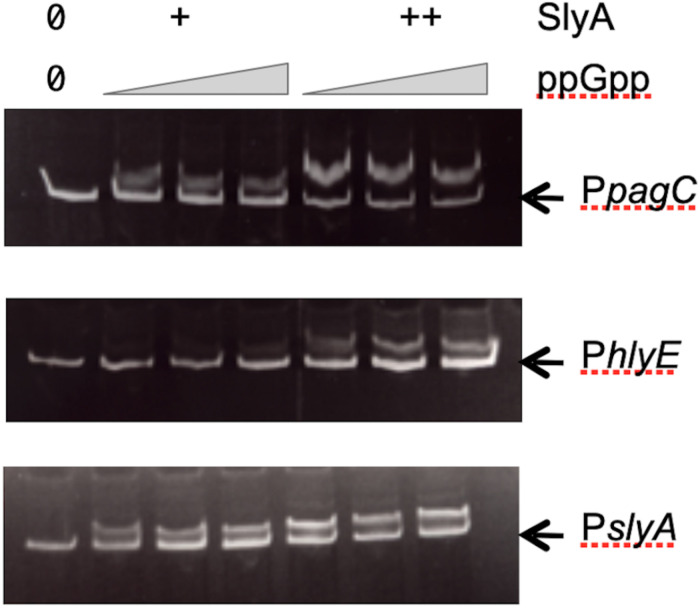
Effect of ppGpp on SlyA binding to DNA. Electrophoretic mobility shift assays were performed using purified SlyA from *E. coli* [at 50 (+) or 100 (++) nM] or purified SlyA from *Salmonella* for the *pagC* experiment [at 200 (+) or 400 (++) nM]. For each SlyA concentration, 0, 50, or 100 μM ppGpp were added.

Our results obtained *in vivo* suggested that ppGpp had no role in SlyA function, contrary to what was reported before (Zhao et al., 2008). Therefore, it was necessary to also test the effect of ppGpp on SlyA DNA binding *in vitro*. Using gel shift assays, we were able to detect a robust binding of SlyA on the promoter regions tested:*hlyE*, *slyA*, and *pagC_Stm* (Supplementary Figure S3A). We then choose for each binding assay, SlyA/DNA ratios that were just sufficient to detect a shift in order to test the effect of adding ppGpp. With addition of 50 μM or 100 μM ppGpp [the same concentrations used by Zhao et al. (2008)], the shifts were not affected (Figure 4). Because we used purified SlyA proteins with a 6his tag fused at the N-terminal, we also performed the same experiments after removing the tag by TEV cleavage. Also, ppGpp might have been trapped with SlyA during the purification, therefore we performed the purifications in a ppGpp null strain and obtained the same negative result (Supplementary Figure S3B).

**FIGURE 5:**
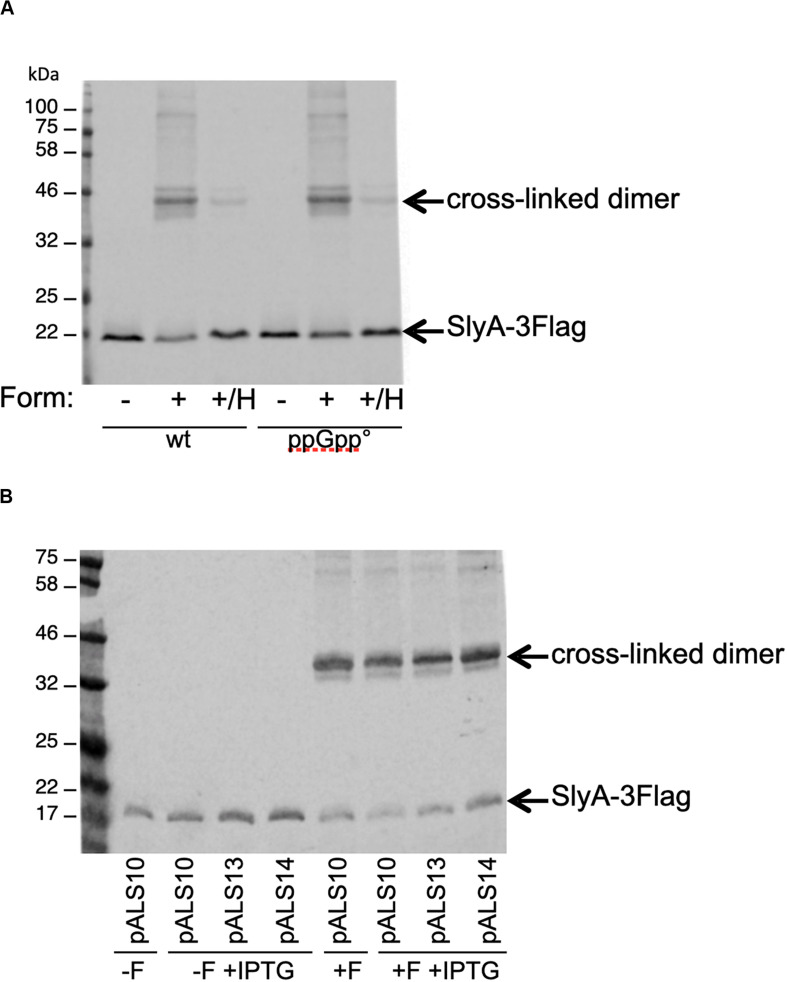
Effect of ppGpp on SlyA dimerization *in vivo*. **(A)** MG1655_SlyA-3Flag (EB1106) and ppGpp°_SlyA-3Flag (EB1110) strains were grown to OD600 = 1.3. **(B)** Strain MG1655_SlyA-3Flag (EB1106) was transformed by plasmids pALS10, pALS13, and pALS14 (pEB0697, pEB0698, and pEB0699, respectively) (Svitil et al., 1993). These transformed strains were grown to OD600 = 1.5 and then *relA* expression was induced with 1 mM IPTG for 30 min. Then, for both panels, the cells were cross-linked with formaldehyde as described in section “Materials and Methods.” +F, with formaldehyde; –F, without formaldehyde; +/H, with formaldehyde and then heated at 96°C. After SDS-PAGE and Western blot, the SlyA-3Flag tag was detected with monoclonal anti-Flag M2.

The last reported effect of ppGpp on SlyA, was that it enhanced its dimerization, as shown by *in vivo* cross-linking experiments (Zhao et al., 2008). In order to detect SlyA by Western blot, we constructed wild type and ppGpp° strains producing a SlyA-3Flag tagged protein expressed from its endogenous locus. SlyA-3Flag was readily detected in the two genetic backgrounds, at the expected size of approximately 20 kDa (Figure 5A). To test the dimerization, we used whole cell cross-linking with formaldehyde. This cross-linker produces covalent bonds that can be destroyed by heating at 96°C. In the wild type background, dimerization of SlyA was clearly detected by cross-linking with formaldehyde (Figure 5A). The dimerization was identical in the ppGpp° background (Figure 5A). In reverse, we decided to test if an excess of ppGpp might affect SlyA dimerization, by overproducing the RelA ppGpp synthase. Plasmids pALS10, pALS13, and pALS14 code, respectively, for a full RelA protein, a constitutively active truncated RelA protein, and an inactive RelA protein (Svitil et al., 1993). We performed the cross-linking experiment in MG1655 strain transformed by these three plasmids and with induction of RelA variants expression. In the samples with induced ppGpp production (pALS10 and pALS13), SlyA dimerization was not affected, or even slightly diminished (Figure 5B). In conclusion, we were not able to see any positive effect of ppGpp on SlyA dimerization.

## Discussion

In this study, we showed that ppGpp is not required for SlyA function in *E. coli*. The expression of several reporter genes was still induced by SlyA overproduction in the absence of ppGpp *in vivo*, SlyA binding to DNA was not improved by adding ppGpp *in vitro*, and finally SlyA dimerization was not affected by ppGpp absence or increased levels *in vivo*. Even if the initial report of ppGpp effect on SlyA is now more than 10 years old (Zhao et al., 2008), we think this information is important and of public good for the community of researchers working on ppGpp. Indeed, we are aware of several groups that were interested in developing ppGpp sensors based on this observation, including ourselves. To our knowledge, no confirmation or disproof of ppGpp effect on SlyA was reported since then, apart from a brief mention that ppGpp had no effect on SlyA control of the *fimB* promoter in *E. coli* (McVicker et al., 2011). Furthermore, SlyA was not spotted in two independent global studies aiming at identifying ppGpp binding proteins (Zhang et al., 2018; Wang et al., 2019). It is therefore still unclear what molecule can regulate SlyA activity. However, recent work provided strong evidence of SlyA control by Salicylate, which fits with SlyA belonging to the MarR family containing proteins known to respond to small aromatic carboxylate compounds (Dolan et al., 2011; Will et al., 2019).

The obvious difference that could explain the discrepancy between our work and the one reported in Zhao et al., 2008 is that we have studied the activity of SlyA in *E. coli*, while the previous study was done in *Salmonella* (Zhao et al., 2008), and that we have studied different reporter genes controlled by SlyA. In particular, the expression of *slyA* itself was a very useful reporter of SlyA action, since it is not dependent on H-NS, and permitted to observe that it was not affected in the absence of ppGpp (Figure 2A). Furthermore, we do not contradict the fact that *pagCD* promoters are shut down in a ppGpp° strain in *Salmonella* as shown in Zhao et al., 2008. We propose that it is in fact very similar to what we observed for the expression of *hlyE* in *E. coli*, for which SlyA overproduction can partially counteract the negative effect of ppGpp absence (Figure 2B), as it was observed for *pagCD* in *Salmonella* (Zhao et al., 2008). Production of SlyA_eco or SlyA_stm had identical effects when produced in *E. coli* (Figure 1B). Inversely, it was shown that production of SlyA_eco in *Salmonella* is able to counter silence the expression of *pagC*, similarly, to SlyA_stm (Will et al., 2019). Therefore, we think the molecular mechanism of SlyA is identical in the two bacteria. However, it is clear that the regulons and the physiological role of SlyA are very different in the two bacteria. This difference does not come from the SlyA protein itself, but from the variations in intergenic and regulatory regions of the target genes. A striking difference is for example that SlyA represses its own expression in *Salmonella* (Stapleton et al., 2002; Will et al., 2019), whereas it auto-activates its expression in *E. coli* as we showed here (Figure 1) and as it was demonstrated before (Corbett et al., 2007). The expression level of *slyA* might also play a role, as it has been suggested that *slyA* expression is much lower in *E. coli* than in *Salmonella* (Will et al., 2019). However, in our experiments, the P*slyA* transcriptional fusion was one of the few to display a robust basal expression level, and we were able to detect the SlyA-3Flag tagged protein expression in *E. coli* (Figure 5). Still, only SlyA overproduction using pBAD-SlyA plasmid permitted to detect expression of *paaA*, *hlyE*, and *pagC*_Stm, suggesting a strong excess of SlyA is necessary to overcome H-NS repression on these genes.

Concerning the effect of ppGpp on SlyA binding to DNA *in vitro*, and the dimerization of SlyA *in vivo*, the discrepancy between our results and the previous ones (Zhao et al., 2008) is more difficult to understand. Indeed, the SlyA proteins of *E. coli* and *Salmonella* are highly similar (91% identical and 95% similar over 142 amino acids), and we performed *in vitro* binding experiments with SlyA proteins purified from both *E. coli* and *Salmonella*, including a binding experiment on a similar *Salmonella pagCD* intergenic region as the one used previously (Figure 4). As described in the result section, we performed several control experiments to rule out any effect of the tag or the purification procedure of SlyA proteins (Supplementary Figure S3B). For the *in vivo* dimerization detected by cross-linking with formaldehyde, we performed the experiment in an *E. coli* strain producing a SlyA-tagged protein expressed from its endogenous locus. Zhao *et al.* performed this experiment in *Salmonella*, with a SlyA-tagged protein expressed from a plasmid. In this case, an indirect effect of ppGpp on *slyA* expression might explain the different results.

The interpretation of the experiments performed in strains mutated for global regulators (such as ppGpp) is complicated by the mode of action of SlyA, which is not a direct and classical activator, but acts mainly as a counter silencer of H-NS. It has been shown that ppGpp physiological effects are intermixed with global regulators such as Fis, CRP, or H-NS, and even DNA supercoiling state (Johansson et al., 2000; Travers and Muskhelishvili, 2005). Therefore, it is to be expected that any tinkering of ppGpp concentrations *in vivo* will affect a complex network of global regulations. Particular promoters such as *pagC* in *Salmonella* or *hlyE* in *E. coli* are controlled by an especially high numbers of specific and global factors, not only H-NS and SlyA, but also PhoPQ and EmrR in the case of *pagC* in *Salmonella* (Zhao et al., 2008; Yang et al., 2019) or CRP and FNR for *hlyE* in *E. coli* (Westermark et al., 2000; Bradley et al., 2007). Obviously, these complex regulatory networks might be affected by ppGpp levels, together with a possible direct effect of ppGpp on the RNAP depending on the nature of the promoter itself (Gourse et al., 2018), as it might be the case for *hlyE* in our study or *pagC* in *Salmonella* (Zhao et al., 2008). More generally, because ppGpp impacts global regulatory networks central to the physiology of bacteria, our study should be taken as a warning of caution in the interpretation of *in vivo* effects triggered by the modification of ppGpp levels.

## Data Availability Statement

All datasets presented in this study are included in the article/Supplementary Material.

## Author Contributions

EB and JV designed the study. EB and JB designed and performed the experiments. EB, JV, and JB discussed the results and wrote the manuscript. All authors contributed to the article and approved the submitted version.

## Conflict of Interest

The authors declare that the research was conducted in the absence of any commercial or financial relationships that could be construed as a potential conflict of interest.

## Abbreviations

*E. coli*: *Escherichia coli*
GFP: green fluorescent protein
ppGpp: guanosine tetraphosphate.

## References

[B1] Baba T. Ara T. Hasegawa M. Takai Y. Okumura Y. Baba M. (2006). Construction of *Escherichia coli* K-12 in-frame, single-gene knockout mutants: the Keio collection. *Mol. Syst. Biol.* 2:2006.0008. 10.1038/msb4100050 PMC1681482 16738554

[B2] Bennison D. J. Irving S. E. Corrigan R. M. (2019). The impact of the stringent response on TRAFAC GTPases and prokaryotic ribosome assembly. *Cells* 8:1313. 10.3390/cells8111313 31653044 PMC6912228

[B3] Bouveret E. Derouiche R. Rigal A. Lloubès R. Lazdunski C. Bénédetti H. (1995). Peptidoglycan-associated lipoprotein-TolB interaction. A possible key to explaining the formation of contact sites between the inner and outer membranes of *Escherichia coli*. *J. Biol. Chem.* 270 11071–11077. 10.1074/jbc.270.19.11071 7744736

[B4] Bradley M. D. Beach M. B. de Koning A. P. J. Pratt T. S. Osuna R. (2007). Effects of Fis on *Escherichia coli* gene expression during different growth stages. *Microbiology* 153 2922–2940. 10.1099/mic.0.2007/008565-0 17768236

[B5] Chalabaev S. Chauhan A. Novikov A. Iyer P. Szczesny M. Beloin C. (2014). Biofilms formed by gram-negative bacteria undergo increased lipid a palmitoylation, enhancing in vivo survival. *mBio* 5:e01116-14. 10.1128/mBio.01116-14 25139899 PMC4147861

[B6] Cherepanov P. P. Wackernagel W. (1995). Gene disruption in *Escherichia coli*: TcR and KmR cassettes with the option of Flp-catalyzed excision of the antibiotic-resistance determinant. *Gene* 158 9–14. 10.1016/0378-1119(95)00193-a 7789817

[B7] Corbett D. Bennett H. J. Askar H. Green J. Roberts I. S. (2007). SlyA and H-NS regulate transcription of the *Escherichia coli* K5 capsule gene cluster, and expression of slyA in *Escherichia coli* is temperature-dependent, positively autoregulated, and independent of H-NS. *J. Biol. Chem.* 282 33326–33335. 10.1074/jbc.m703465200 17827501

[B8] Curran T. D. Abacha F. Hibberd S. P. Rolfe M. D. Lacey M. M. Green J. (2017). Identification of new members of the *Escherichia coli* K-12 MG1655 SlyA regulon. *Microbiology* 163 400–409. 10.1099/mic.0.000423 28073397 PMC5797941

[B9] Dalebroux Z. D. Svensson S. L. Gaynor E. C. Swanson M. S. (2010). ppGpp conjures bacterial virulence. *Microbiol. Mol. Biol. Rev.* 74 171–199. 10.1128/mmbr.00046-09 20508246 PMC2884408

[B10] Datsenko K. A. Wanner B. L. (2000). One-step inactivation of chromosomal genes in *Escherichia coli* K-12 using PCR products. *Proc. Natl. Acad. Sci. U.S.A.* 97 6640–6645. 10.1073/pnas.120163297 10829079 PMC18686

[B11] Dolan K. T. Duguid E. M. He C. (2011). Crystal structures of SlyA protein, a master virulence regulator of *Salmonella*, in free and DNA-bound states. *J. Biol. Chem.* 286 22178–22185. 10.1074/jbc.m111.245258 21550983 PMC3121362

[B12] Ellison D. W. Miller V. L. (2006). Regulation of virulence by members of the MarR/SlyA family. *Curr. Opin. Microbiol.* 9 153–159. 10.1016/j.mib.2006.02.003 16529980

[B13] Gourse R. L. Chen A. Y. Gopalkrishnan S. Sanchez-Vazquez P. Myers A. Ross W. (2018). Transcriptional responses to ppGpp and DksA. *Annu. Rev. Microbiol.* 72 163–184. 10.1146/annurev-micro-090817-062444 30200857 PMC6586590

[B14] Guzman L. M. Belin D. Carson M. J. Beckwith J. (1995). Tight regulation, modulation, and high-level expression by vectors containing the arabinose PBAD promoter. *J. Bacteriol.* 177 4121–4130. 10.1128/jb.177.14.4121-4130.1995 7608087 PMC177145

[B15] Hobbs J. K. Boraston A. B. (2019). (p)ppGpp and the stringent response: an emerging threat to antibiotic therapy. *ACS Infect. Dis.* 5 1505–1517. 10.1021/acsinfecdis.9b00204 31287287

[B16] Johansson J. Balsalobre C. Wang S. Y. Urbonaviciene J. Jin D. J. Sondén B. (2000). Nucleoid proteins stimulate stringently controlled bacterial promoters: a link between the cAMP-CRP and the (p)ppGpp regulons in *Escherichia coli*. *Cell* 102 475–485. 10.1016/s0092-8674(00)00052-0 10966109

[B17] Karp P. D. Ong W. K. Paley S. Billington R. Caspi R. Fulcher C. (2018). The EcoCyc database. *EcoSal Plus* 8:10.1128/ecosalplus.ESP-0009-2013. 10.1128/ecosalplus.ESP-0009-2013 30406744 PMC6504970

[B18] Libby S. J. Goebel W. Ludwig A. Buchmeier N. Bowe F. Fang F. C. (1994). A cytolysin encoded by *Salmonella* is required for survival within macrophages. *Proc. Natl. Acad. Sci. U.S.A.* 91 489–493. 10.1073/pnas.91.2.489 8290552 PMC42974

[B19] Lithgow J. K. Haider F. Roberts I. S. Green J. (2007). Alternate SlyA and H-NS nucleoprotein complexes control hlyE expression in *Escherichia coli* K-12. *Mol. Microbiol.* 66 685–698. 10.1111/j.1365-2958.2007.05950.x 17892462 PMC2156107

[B20] McVicker G. Sun L. Sohanpal B. K. Gashi K. Williamson R. A. Plumbridge J. (2011). SlyA protein activates fimB gene expression and type 1 fimbriation in *Escherichia coli* K-12. *J. Biol. Chem.* 286 32026–32035. 10.1074/jbc.m111.266619 21768111 PMC3173223

[B21] Navarre W. W. Halsey T. A. Walthers D. Frye J. McClelland M. Potter J. L. (2005). Co-regulation of *Salmonella enterica* genes required for virulence and resistance to antimicrobial peptides by SlyA and PhoP/PhoQ. *Mol. Microbiol.* 56 492–508. 10.1111/j.1365-2958.2005.04553.x 15813739

[B22] Potrykus K. Cashel M. (2008). (p)ppGpp: still magical. *Annu. Rev. Microbiol.* 62 35–51. 10.1146/annurev.micro.62.081307.162903 18454629

[B23] Stapleton M. R. Norte V. A. Read R. C. Green J. (2002). Interaction of the *Salmonella* Typhimurium transcription and virulence factor SlyA with target DNA and identification of members of the SlyA regulon. *J. Biol. Chem.* 277 17630–17637. 10.1074/jbc.m110178200 11882648

[B24] Stoebel D. M. Free A. Dorman C. J. (2008). Anti-silencing: overcoming H-NS-mediated repression of transcription in Gram-negative enteric bacteria. *Microbiology* 154 2533–2545. 10.1099/mic.0.2008/020693-0 18757787

[B25] Studier F. W. Moffatt B. A. (1986). Use of bacteriophage T7 RNA polymerase to direct selective high-level expression of cloned genes. *J. Mol. Biol.* 189 113–130. 10.1016/0022-2836(86)90385-2 3537305

[B26] Svitil A. L. Cashel M. Zyskind J. W. (1993). Guanosine tetraphosphate inhibits protein synthesis in vivo. A possible protective mechanism for starvation stress in *Escherichia coli*. *J. Biol. Chem.* 268 2307–2311. 8428905

[B27] Travers A. Muskhelishvili G. (2005). DNA supercoiling - a global transcriptional regulator for enterobacterial growth. *Nat. Rev. Microbiol.* 3 157–169. 10.1038/nrmicro1088 15685225

[B28] Wahl A. My L. Dumoulin R. Sturgis J. N. Bouveret E. (2011). Antagonistic regulation of dgkA and plsB genes of phospholipid synthesis by multiple stress responses in *Escherichia coli*. *Mol. Microbiol.* 80 1260–1275. 10.1111/j.1365-2958.2011.07641.x 21463370

[B29] Wang B. Dai P. Ding D. Del Rosario A. Grant R. A. Pentelute B. L. (2019). Affinity-based capture and identification of protein effectors of the growth regulator ppGpp. *Nat. Chem. Biol.* 15 141–150. 10.1038/s41589-018-0183-4 30559427 PMC6366861

[B30] Westermark M. Oscarsson J. Mizunoe Y. Urbonaviciene J. Uhlin B. E. (2000). Silencing and activation of ClyA cytotoxin expression in *Escherichia coli*. *J. Bacteriol.* 182 6347–6357. 10.1128/jb.182.22.6347-6357.2000 11053378 PMC94780

[B31] Will W. R. Brzovic P. Le Trong I. Stenkamp R. E. Lawrenz M. B. Karlinsey J. E. (2019). The evolution of SlyA/RovA transcription factors from repressors to countersilencers in *Enterobacteriaceae*. *mBio* 10:e00009-e19. 10.1128/mBio.00009-19 30837332 PMC6401476

[B32] Will W. R. Fang F. C. (2020). The evolution of MarR family transcription factors as counter-silencers in regulatory networks. *Curr. Opin. Microbiol.* 55 1–8. 10.1016/j.mib.2020.01.002 32044654 PMC7311280

[B33] Will W. R. Navarre W. W. Fang F. C. (2015). Integrated circuits: how transcriptional silencing and counter-silencing facilitate bacterial evolution. *Curr. Opin. Microbiol.* 23 8–13. 10.1016/j.mib.2014.10.005 25461567 PMC4323728

[B34] Wyborn N. R. Stapleton M. R. Norte V. A. Roberts R. E. Grafton J. Green J. (2004). Regulation of *Escherichia coli* hemolysin E expression by H-NS and *Salmonella* SlyA. *J. Bacteriol.* 186 1620–1628. 10.1128/jb.186.6.1620-1628.2004 14996792 PMC355951

[B35] Yang D. Kong Y. Sun W. Kong W. Shi Y. (2019). A dopamine-responsive signal transduction controls transcription of *Salmonella enterica* serovar typhimurium virulence genes. *mBio* 10:e02772-18. 10.1128/mBio.02772-18 30992361 PMC6469979

[B36] Zaslaver A. Bren A. Ronen M. Itzkovitz S. Kikoin I. Shavit S. (2006). A comprehensive library of fluorescent transcriptional reporters for *Escherichia coli*. *Nat. Methods* 3 623–628. 10.1038/nmeth895 16862137

[B37] Zeghouf M. Li J. Butland G. Borkowska A. Canadien V. Richards D. (2004). Sequential peptide affinity (SPA) system for the identification of mammalian and bacterial protein complexes. *J. Proteome Res.* 3 463–468. 10.1021/pr034084x 15253427

[B38] Zhang Y. Zborníková E. Rejman D. Gerdes K. (2018). Novel (p)ppGpp binding and metabolizing proteins of *Escherichia coli*. *mBio* 9:e02188-17. 10.1128/mBio.02188-17 29511080 PMC5845004

[B39] Zhao G. Weatherspoon N. Kong W. Curtiss R. Shi Y. (2008). A dual-signal regulatory circuit activates transcription of a set of divergent operons in *Salmonella* Typhimurium. *Proc. Natl. Acad. Sci. U.S.A.* 105 20924–20929. 10.1073/pnas.0807071106 19091955 PMC2634881

