# Supplementary figures and images for: SlyA Transcriptional Regulator Is Not Directly Affected by ppGpp Levels
Source: Front Microbiol. 2020 Aug 4;11:1856. doi: 10.3389/fmicb.2020.01856 (PMC7417354; doi:10.3389/fmicb.2020.01856)

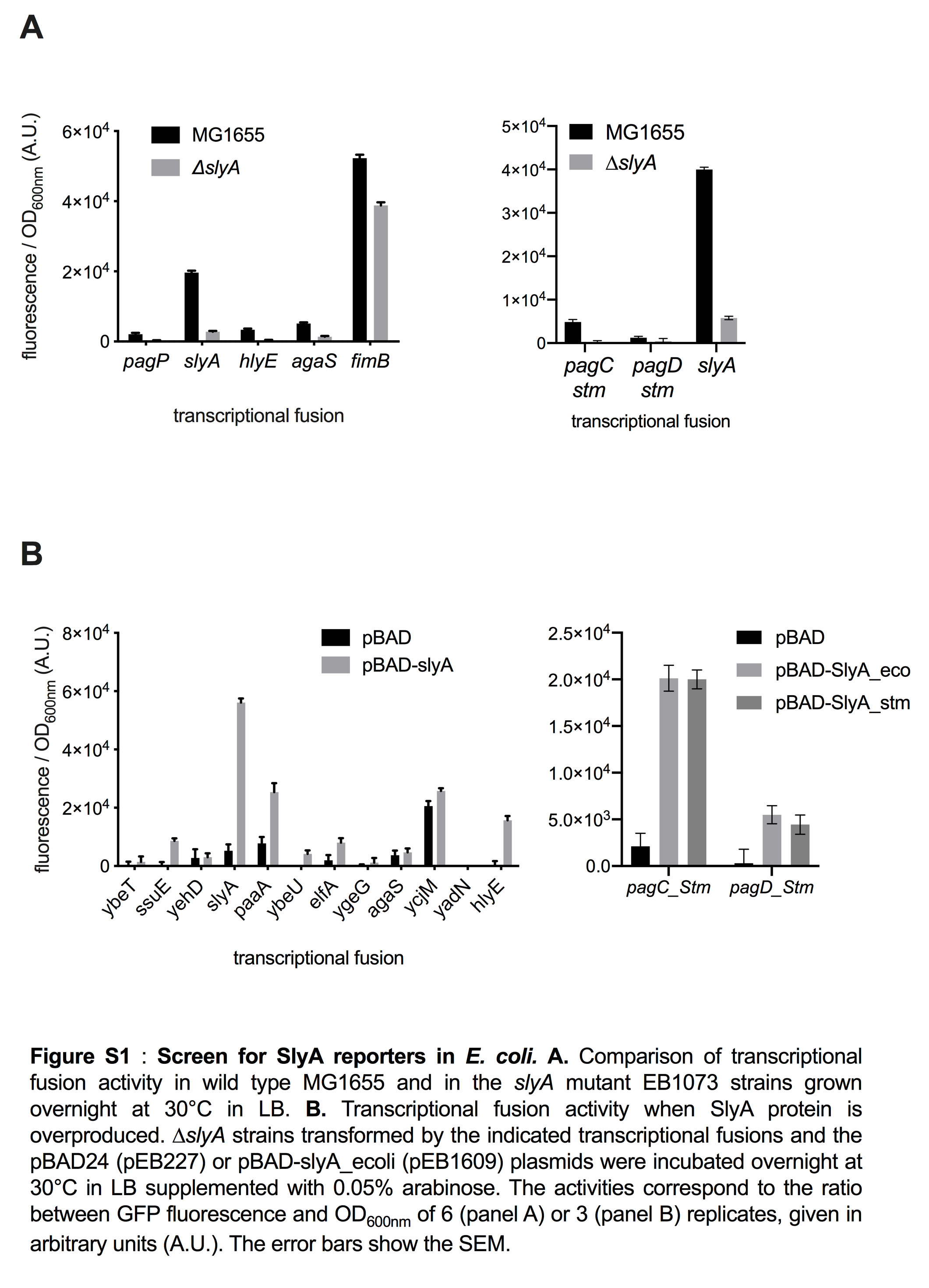

Supplement: Supplementary file 1 [file Image_1.TIFF]

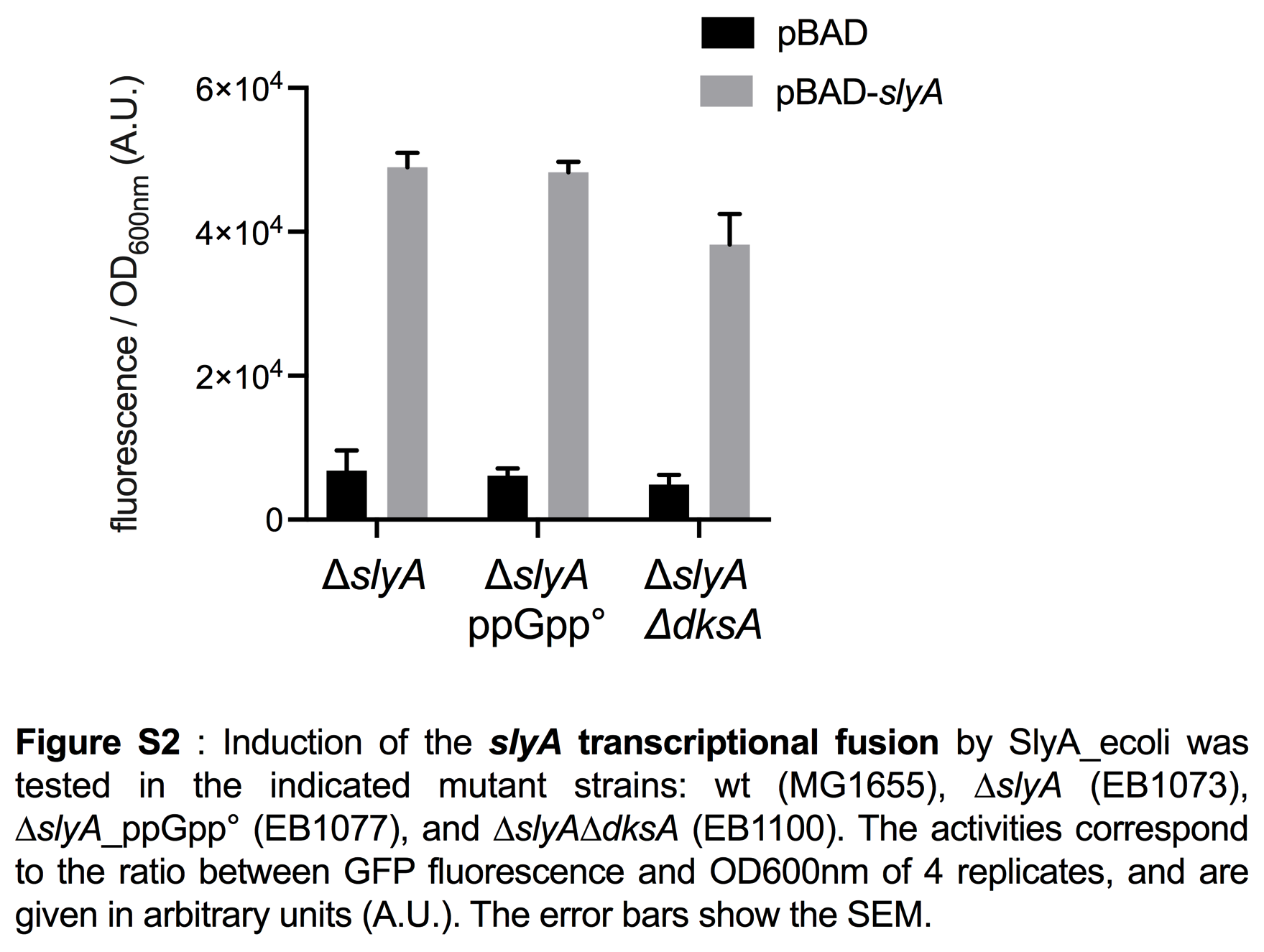

Supplement: Supplementary file 2 [file Image_2.TIFF]

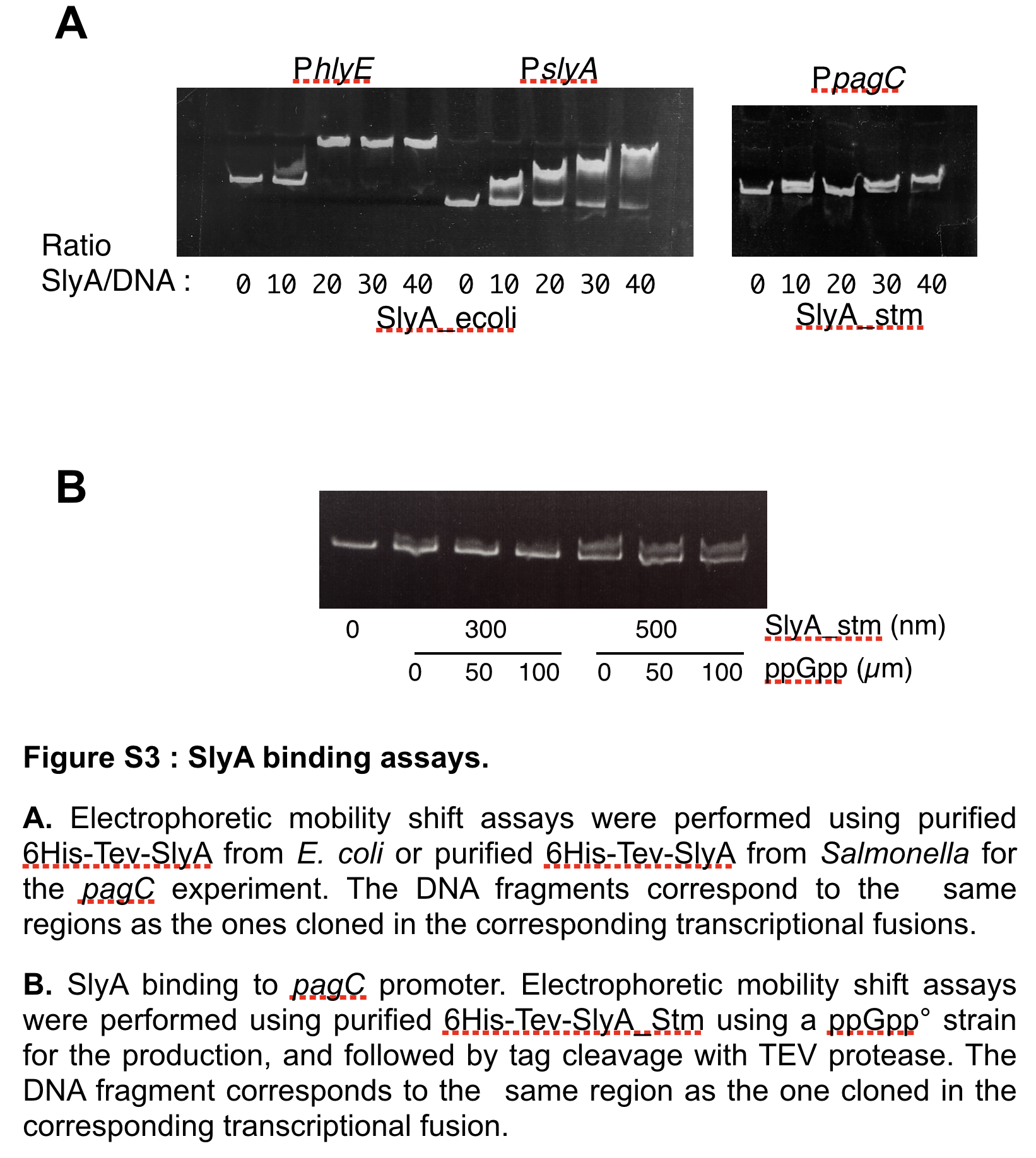

Supplement: Supplementary file 3 [file Image_3.TIFF]
